# Graphene Oxide Aerosol Deposition and its Influence on Cancer Cells. Preliminary Results

**DOI:** 10.3390/ma13194464

**Published:** 2020-10-08

**Authors:** Barbara Nasiłowska, Zdzisław Bogdanowicz, Kinga Hińcza, Zygmunt Mierczyk, Stanisław Góźdź, Małgorzata Djas, Krystian Kowiorski, Aneta Bombalska, Artur Kowalik

**Affiliations:** 1 Institute of Optoelectronics, Military University of Technology, gen. S. Kaliskiego 2, 00-908 Warsaw, Poland; zygmunt.mierczyk@wat.edu.pl (Z.M.); aneta.bombalska@wat.edu.pl (A.B.); 2 Faculty of Mechanical Engineering, Military University of Technology, gen. S. Kaliskiego 2, 00-908 Warsaw, Poland; zdzislaw.bogdanowicz@wat.edu.pl; 3 Department of Molecular Diagnostics, Holy Cross Cancer Center, Kielce, S. Artwińskiego 3, 25-735 Kielce, Poland; Kinga.Hincza@onkol.kielce.pl (K.H.); Artur.Kowalik@onkol.kielce.pl (A.K.); 4 Department of Clinical Oncology, Holy Cross Cancer Center, Kielce, S. Artwińskiego 3, 25-735 Kielce, Poland; Stanislaw.Gozdz@onkol.kielce.pl; 5 Department of Prophylaxis and Cancer Epidemiology, Collegium Medicum, Jan Kochanowski University, Al. IX Wieków Kielc 19A, 25-317 Kielce, Poland; 6 Łukasiewicz Research Network—Institute of Electronic Materials Technology, Department of Chemical Synthesis and Flake Graphene; Wólczyńska 133, Warsaw 01-919, Poland; malgorzata.djas@itme.edu.pl (M.D.); krystian.kowiorski@itme.edu.pl (K.K.); 7 Division of Medical Biology, Institute of Biology Jan Kochanowski University, Uniwersytecka 7, 25-406 Kielce, Poland

**Keywords:** graphene oxide, cancer cells, GO aerosol, deposition

## Abstract

This paper presents the results of the interaction of graphene oxide (GO) on MDA-MB-231 and SW-954 cancer cell lines. The tests were carried out in two variants. In the first one, GO was sprayed on a Petri dish and then, the cancer cell lines were cultured. In the second variant, the cells were covered with an aerosol containing GO. In both variants, cancer cell lines were incubated and tested every 24, 48, and 72 h. After each time period, cell viability and surface morphology were measured. The tests after 72 h showed that coating with GO aerosol caused a reduction in cell viability by 52.7% and 26.4% for MDA-MB-231 and SW-954 cancer cell lines, respectively, with respect to a reference sample (without the influence of GO aerosol). Tests where GO is a culture medium demonstrated a decrease in cell viability by approximately 4.3% compared to a reference sample for both considered cell lines.

## 1. Introduction

Since 2010, when A. Geim and K. Novoselov were awarded a Nobel Prize in physics for graphene discovery, a great deal of research has been carried out concerning graphene and its derivatives [1]. Researchers have searched for different graphene applications in electronics [2,3,4,5], informatics [6], construction [7], material engineering [8,9,10,11,12,13], mechanics [10], medicine [11], and even sport [12].

Up to this day, numerous methods of graphene, reduced graphene oxide (rGO), and graphene oxide (GO) deposition have been developed. One of the most effective is chemical vapor deposition (CVD) [14,15,16,17]. One interesting method of thin layer deposition is the spray method. The first to examine and develop this method were Do-Yeon Kim et al. [18]. They presented supersonic kinetic spraying as a method for rGO deposition on any kind of substrate. Graphene deposition with the use of this method turned out to be less complicated, more economical, and allowed large-scale graphene deposition [18]. Some papers reported graphene and GO deposition in terms of corrosion resistance [19,20] properties and improvement of electric [21] and material [22] properties. When analyzing medical applications of graphene, one can find reports about cell [23] and bacterial cytotoxicity [24,25].

Sengupta et al. [25] showed GO and rGO influence Gram-positive and Gram-negative bacteria. They reported that graphene/graphene oxide (G/GO) antibacterial activity depends on size, shape, and the kind of bacteria examined. GO was found to be more effective due to its oxygen functional groups. GO showed the ability to suppress cellular growth by inducing oxidative stress which destabilizes cell membranes and leads to cytoplasm leakage [25,26,27].

Jaworski et al. [28] reported results concerning the influence of graphene platelets (GPs) on the morphology and viability of glioma cells. It was found that graphene is toxic to glioma cells and induces apoptosis in the U188 cell line. No necrosis was reported. Glioma cells have the highest degree of malignancy, migrate intensely and fast, and spread fast in brain tissues. It was observed that the cytotoxic effect was proportional to the graphene concentration. Microscopic studies showed that graphene platelets (GPs) adhered to the cells, forming a thin and impermeable layer. This layer surrounds the body of the glioma cells, separates them from the extracellular environment, blocks the transport of various substances into the cell, and influences respiratory processes.

Tabish T.A. et al. [29] presented the results of functionalized graphene oxide, which during decomposition, adsorbed the enzymes responsible for invasiveness and tumor metastasis formation. Due to the cationic and hydrophilic residues of GO, enzymes such as Cathepsin D and Cathepsin L (CasthD and CathL) were bound on the GO surface. Additionally, Tabish et al. found that the GO interaction led to the apoptosis of cancer cells. Wang X. et al. [30], in their publication, investigated the effect of GO on the ability of glioblastoma stem-like cells (GSCs) to form spheres, cell divisions, viability, and differentiation. They found that GO inhibits cell division and the formation of cell spheres through epigenetic mechanisms responsible for cell differentiation, reducing their ability to proliferate. Pei et al. [31] proved that GO nanoparticles are not toxic to cells, but the addition of PEGylated nano-graphene oxide to cisplatin (Pt) and doxorubicin (DOX) anticancer drugs resulted in apoptosis and necrosis of cancer cells, but also in a reduction in tumor size in vivo.

In the recent literature, inconsistent results are described concerning the influence of graphene and its derivatives on cells and tissues [32]. Some indicate biocompatibility [33,34] and others report that graphene flakes can cut through cells and damage them [35,36,37].

Undoubtedly, GO cannot be treated as the same kind of material. Their properties depend on three basic parameters: the number of graphene layers, surface development, and relation of carbon to oxygen atoms [38,39]. The fabrication method of graphene and its derivatives is crucial. Results presented by Jaworski and colleagues [40] showed that rGO, as compared to GO, showed a higher toxic effect on cancer cells. The authors point out that biocompatibility can be influenced by the purity of graphene after fabrication [40] and its concentration [39,40].

Interesting results were presented by Zhu Y. and coworkers [35]. They reported that cell membrane damage of human keratinocytes, epithelium lung cells, and murine macrophages progressed along the irregular edge of graphene flakes or sharp edges.

Such properties are the result of graphene structure, mainly sharp edges, when graphene or GO flakes are applied. Sharp edges cause physical damage to the cell membrane, which results in loss of membrane integrity and the leakage of intracellular content leading to its death.

The question occurs as to what kind of influence GO has on cancer cells. In the literature, the hypothesis that GO deposited on the cell surface can snip cell off air and nutrients leading to apoptosis is also described.

Due to its high surface oxygen content, GO causes oxidative stress and in this way, induces cellular death [36].

Inspired by the diversity and complexity of the problems described by these authors, it was attempted in this study to deposit GO in aerosol form on breast and vulva cancer cells and to process their culture. The results in the literature were compared with those described below. Selected cancer cell lines were cultured on a Petri dish with GO deposited on it. The authors realize that results indicating lowering the cancer cells’ viability have tremendous significance in oncology focused on GO deposition in a form of aerosol.

The application of GO in oncology gives new possibilities in anticancer treatments. To the best of our knowledge, there is no other report dealing with GO spray and its influence on cancer cells. This article, therefore, presents insight into the possibility of new methods for cancer treatment.

## 2. Methodology

**Figure 1:**
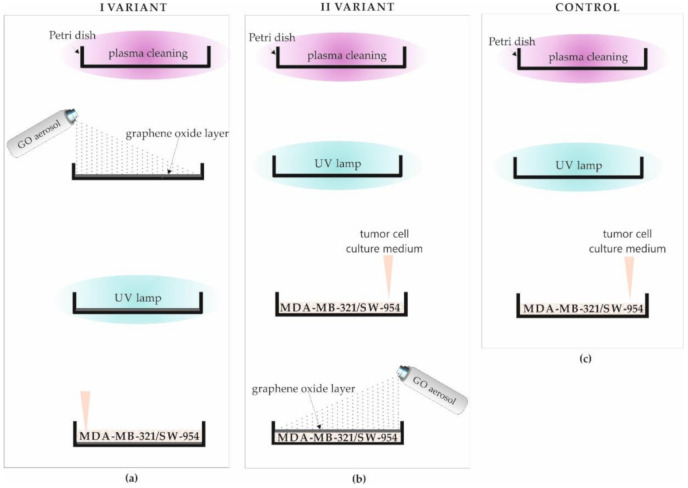
Scheme of preparation of Petri dishes for cancer cell culture in: (**a**) I VARIANT—Petri dish plasma cleaning, GO deposition, sterilization of the Petri dish surface with a UV lamp, and cells culturing; (**b**) II VARIANT—Petri dish plasma cleaning, sterilization of the Petri dish surface with a UV lamp, cell culturing, and GO deposition; (**c**) CONTROL—Petri dish plasma cleaning, sterilization of the Petri dish surface with UV lamp, and cells culturing.

The experiments, aiming to examine the influence of GO on breast and vulva cancer cells, were carried out in two variants and control:

1. GO was deposited on the plasma cleaned Petri dish and then, it was subject to a vacuum drying process. The next step was sterilization with UV rays. The authors developed a procedure of depositing the GO on the glass. In that procedure, GO was sprayed under pressure, and then, it was subjected to vacuum drying at a temperature of 150 °C (Figure 1a).
2. The plasma cleaned Petri dish was exposed to UV rays as a sterilization process. MDA-MB-231 and SW-954 cancer cell lines were cultured on a prepared Petri dish and then, coated with GO in the form of an aerosol (Figure 1b).
3. The reference samples (control) were made similar to variant II for both cell lines, but without the final stage of the GO deposition. The Petri dish was plasma cleaned, subjected to UV radiation for sterilization, and then, the cell culture procedure was performed as it was described in Section 2.3.

A comparative analysis was also carried out with respect to the control sample (control, Figure 1c). The control sample included all procedures performed for variant I and II (plasma cleaning, UV sterilization, and cell culturing) except GO deposition. In the first step of preliminary sample preparation, all Petri dishes were subjected to surface cleaning by plasma reaction for 60 min with a plasma power of 100 W (SPI Supplies Plasma Prep III Etcher, Solid State Design) [41].

Sterilization of the dishes was carried out for 30 min with the use of UV rays at a power of 15 W in a laminar chamber type II safety class DONSERV LA2-5A1-E type.

### 2.1. Materials

**Figure 2:**
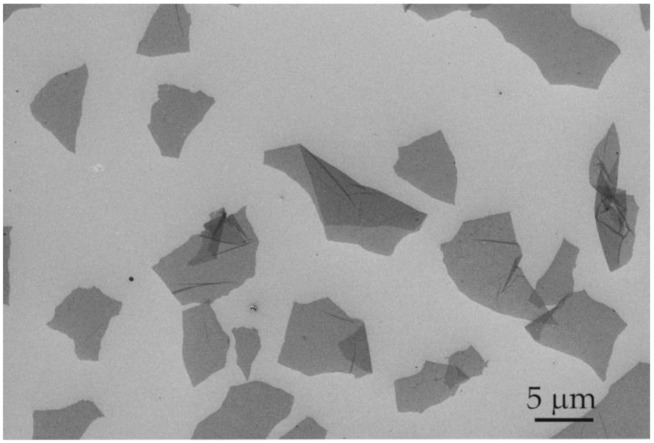
SEM images of GO flakes.

GO flakes (Figure 2) dispersed in water were obtained from flake graphite by a modified Hummers method (Department of Chemical Synthesis and Flake Graphene, Łukasiewicz Research Network—Institute of Electronic Materials Technology, Warsaw, Poland). The average GO thickness was 3–4 layers (around 1.0–1.5 nm) and flake size was 3–10 µm, and the oxygen content was 45–52% [42]. Flake graphite (Asbury Carbons, Asbury, NJ, USA) was added to a reactor containing concentrated sulfuric acid (H_2_SO_4_, Chempur, Piekary Śląskie, Poland) and phosphoric acid (H_3_PO_4_, Chempur, Piekary Śląskie, Poland). Next, potassium permanganate (KMnO_4_, Chempur, Piekary Śląskie, Poland) was slowly added to this mixture. The oxidation process was performed for a few hours and stopped by the addition of deionized water and hydrogen peroxide (30% H_2_O_2_, Chempur, Piekary Śląskie, Poland). The graphite oxide water suspension obtained was left for sedimentation. Then, the purification and exfoliation processes were performed. The concentration of GO water dispersion was 10 g/L.

### 2.2. GO Aerosol

A GO aerosol was made according to Bag-On-Valve technology (BOV), which allows the application of the suspension of GO in the form of aerosol without propellant gas contact. An aerosol valve BOV Crimper type was connected with a bag made of four-layered foil (polyethylene, aluminum, nylon, and polypropylene). The set included an Under-The-Cup pre-gassing (UTC) part and a BOV valve locking the aluminum container. Sealing the container allowed air to be applied pressure ranging from 1 to 3 bars between pouch and container. In a further step, the pouch was filled with GO suspension up to 9 bars. The aerosol set was equipped with a valve and spray applicator from Aptara. Spraying strength was regulated with air pressure and the applicator nozzle type. The application time of GO on the Petri dish (variant I) and cells (variant II) was the same, so we conclude that the concentration of GO (10 g/L) is identical in both variants.

### 2.3. Cell Culture

The cell lines (MDA-MB-231 and SW-954) used in this study were obtained from the American Type Culture Collection (ATCC) in Manassas, VA, USA.

Experiments concerning the interaction of GO on cancer cells were carried out with the use of two cancer cell lines, i.e., MDA-MB-231 and SW-954. Cancer cells of the MDA-MB-231 line come from a triple-negative breast cancer characterized by a lack of estrogen, progesterone, and HER2 receptors. Cells were cultivated in Falcon T25 cell culture flasks in the presence of Dulbecco’s Minimum Essential Medium Eagle (DMEM) cell culture medium enriched with a 10% fetal bovine serum (FBS) with the addition of selected antibiotics (penicillin/streptomycin). Cancer cells of the SW-954 vulva squamous cell cancer line were cultivated with the use of a L-15 (Leibovitz) medium in the presence of 20% fetal bovine serum (FBS) and antibiotics (penicillin/streptomycin). Both cell lines were cultured under sterile conditions in the incubator in humid conditions containing 5% CO_2_/95% air (MDA-MB-231) and 100% air (SW-954).

In order to obtain cancer cells for the tests described in the paper, a cancer cells line passage procedure was conducted. First, the cell culture medium was poured out, then the cells were rinsed with PBS without Ca^2+^ and Mg^2+^ ions and treated with 0.25% trypsin solution. After separation of cells from the cell culture flask surface, a medium with serum was added to the cell culture flask in order to inactivate the trypsin. The content of the cell culture flask was transferred to a 15 mL test tube and centrifuged (RCF = 300× *g* for 5 min (4 °C)). After that, the supernatant was removed, and the residue was suspended in a fresh medium and used for further tests.

### 2.4. Cell Viability Test

A cancer cell viability test was carried out with the use of trypan blue. The test is based on the use of the natural properties of the cell membrane as barriers for compounds with an anionic nature. Anionic compounds (dyes) do not penetrate living cells due to the negative charge of the cell membrane. In the case of permanent damage to the cell membrane, which takes place after the death of the cell, the membrane potential disappears, which in turn allows the penetration of anionic substances into the cell and staining of the cytoplasm or nucleus.

First, the cells were passaged on Petri dishes. The medium from the above the cells was poured out into Eppendorf tubes and then, the cells were rinsed with warm PBS without Ca^2+^ and Mg^2+^ ions. The cells were separated from the Petri dishes by treating them with a trypsin solution, which was subsequently inactivated with the medium from Eppendorf tubes. Next, the cells were centrifuged (RCF = 300× *g* for 5 min) and the cellular pellet was suspended in 300 μL of fresh medium. Then, a mixture consisting of 10 μL of the cell’s suspension and 10 μL of 0.2% solution of trypan blue was incubated for 2 min and placed in a Bürker chamber in order to calculate the number of living cells. 

Cells of MDA-MB-231 and SW-954 cancer lines (in both variants) were cultured in the incubator in 37 °C under humid conditions containing 5% CO_2_/95% air (MDA-MB-231) and 100% air (SW-954).

The changes in cell viability for variants I and II in comparison with the control samples were presented as percentages. For statistical purposes, cell cultures were replicated 9 times for each cell line, exposure time, and variant.

### 2.5. FTIR Measurements

Measurements of absorption spectra of GO in the infrared range were made using the FTIR–ATR technique. The measurements were made to confirm GO deposition on the Petri dish surface. For ATR measurements, a Thermo Scientific IS50 ATR Module (ThermoFisher SCIENTIFIC, Waltham, MA, USA) was used. Diamond crystal with incident light radiation at 45° was applied. Spectra were recorded three times each with 126 scans in a range of 4000–650 cm^−1^ with a resolution of 4 cm^−1^.

### 2.6. Sample Preparation for Imaging by Scanning Electron Microscopy

For analysis of the surface of the Petri dish, GO deposition characteristics, and collection of the images of cancer cell lines, a Scanning Electron Microscopy (SEM) was used (Quanta 250 FEG SEM, FEI, Hillsboro, OR, USA). In order to improve the conductibility and the quality of SEM images of cancer cells, the samples were dried in a vacuum dryer Vacucell 22 L (BMT Medical Technology s.r.o., Brno-Zábrdovice, Czech Republic) and afterwards, deposited with a 5.04 nm layer of gold with the use of a high vacuum sputtering EM ACE 600. During sputtering, the microscope table rotated under 120°. The gold layer acted not only as a conductor but also provided a protective layer against damage from the electron beam. A SEM image was acquired using a backscattered detector (ETD-BSE, FEI, Hillsboro, OR, USA) with an accelerating voltage of 5 kV for GO and 10 kV for cancer cells (all variants). SEM images analysis established the kind and range of the damage that arose as an interaction between cells and GO aerosol.

### 2.7. Characterization of Cancer Cells by Optical Microscopy

Microscopic observations (mag. 100 and 1000) of the surface morphology were carried out every 24, 48, and 72 h with the use of an optical microscope to assess the influence of GO on the cancer cell morphology. In addition to the assessment of the effect of GO on the morphology of tumor cells, vitality tests were also performed to determine the effect of GO in both variants on the vitality of cells. Performing the viability test allowed the natural state of the cell membrane to be determined and the metabolic state of the cells to be measured, which indicates the potential ability of cells to grow, divide, and metabolize.

### 2.8. Confocal Microscopy

The measurements of the changes in surface roughness as a result of plasma cleansing, UV light interaction, and GO deposition were made using a confocal microscope Zeiss LSM 700 (Carl Zeiss Microscopy, Jena, Germany). The experimental parameters were laser wavelength 405 nm, pinhole 0,5 AU, and gain 416. Ten measurements for each sample were made.

## 3. Results and Discussions

### 3.1. Surface Morphology

The main reason for plasma cleansing was to prepare the surface for GO deposition. This process allowed GO to strongly adsorb on the Petri dish surface [43] and minimized the peeling back of GO flakes during cell culturing. Vacuum drying was applied to reduce the air leftovers trapped between the Petri dish surface and GO layer.

Since other forms of sterilization could considerably influence the GO structure or even peel it off completely, sterilization with UV light was applied. Both processes (drying and sterilization) were carried out to preserve and sterilize the surface of the Petri dish with GO layers and prepare it for the next steps of the experiments.

**Figure 3:**
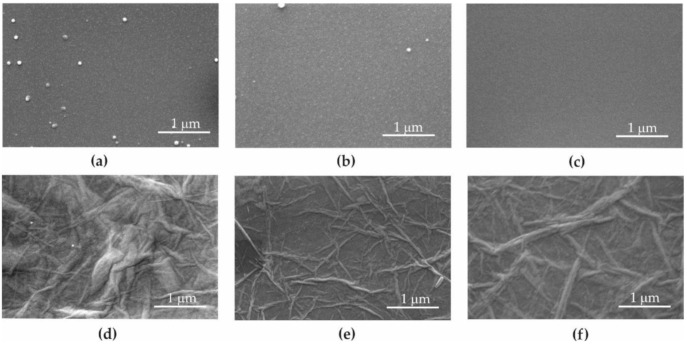
SEM images of the surface. (**a**) Unmodified Petri dish; (**b**) after plasma cleansing; (**c**) plasma cleansing and UV sterilization; (**d**) plasma cleansing and GO deposition; (**e**) plasma cleansing, GO deposition, and vacuum dryer; (**f**) plasma cleansing GO deposition, vacuum dryer, and UV sterilization.

Figure 3 compares the Petri dish topography before (Figure 3a) and after (Figure 3b) plasma cleansing, and after UV sterilization (Figure 3c) and GO aerosol deposition (Figure 3c). Reduction in contamination was observed on the surface of the Petri dish after plasma cleansing and UV sterilization (Figure 3a–c).

Figure 3e shows the changes after vacuum dryer usage and Figure 3f shows the influence of UV radiation on GO deposited on the Petri dish.

As a result of GO deposition in aerosol form, the flake edges overlapped with each other and the bottom of the Petri dish was covered with GO at a level of 100%. Typical characteristics for GO unevenness of the surface were recorded (Figure 3d). The deposition of GO caused an increase in surface roughness <15% (Table 1).

**Figure 4:**
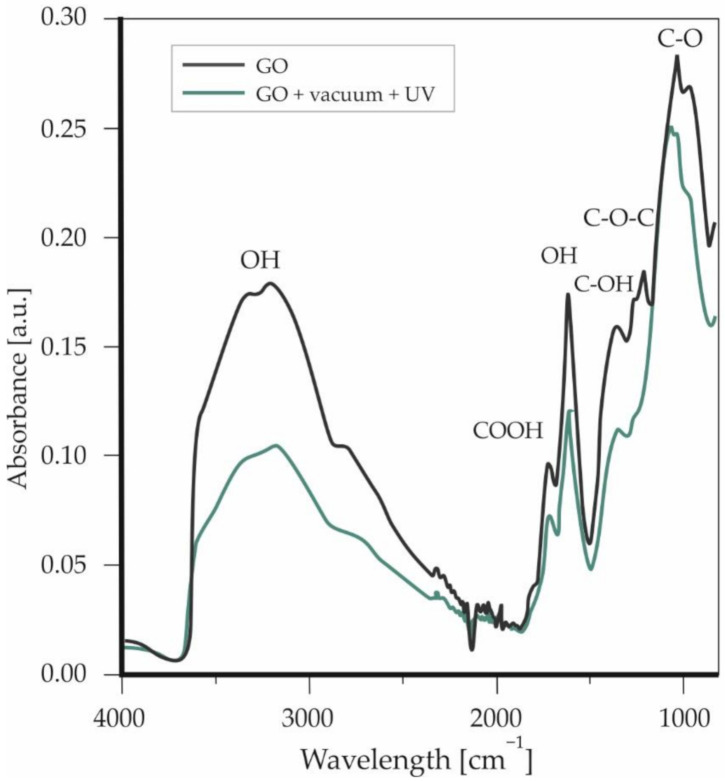
FTIR spectrum of GO deposited on the Petri dish.

In order to confirm the structure of GO after the drying process and sterilization, a FTIR spectrum was recorded (Figure 4). Analysis of the spectrum showed typical absorption bands characteristic for GO.

In a range of 3550–3200 cm^−1^, stretching vibrations originating from OH groups can be observed. The band at 1730 cm^−1^ was assigned to the carboxyl group; 1619 cm^−1^ corresponds to the stretching and bending vibration of OH groups of water molecule adsorbed on GO. The peak at 1365 cm^−1^ arises from C–OH, 1225 cm^−1^, and denotes C–O–C stretching, and 1047 cm^−1^ was assigned to C–O vibrations. The GO spectrum after vacuum drying and UV did not differ drastically from the GO spectrum recorded before these processes. Loss in OH groups is visible through the whole spectrum. This may be the effect of water evaporation from the GO or slight reduction in other OH (along with COOH) groups located in GO structure.

### 3.2. Microscopic Analysis of Cancer Cells

Both cell lines were characterized by a different morphology and growth rate. MDA-MB-231 cells grew faster and the cells were not only larger and more branched but also had longer protrusions than the SW-954 cells.

**Figure 5:**
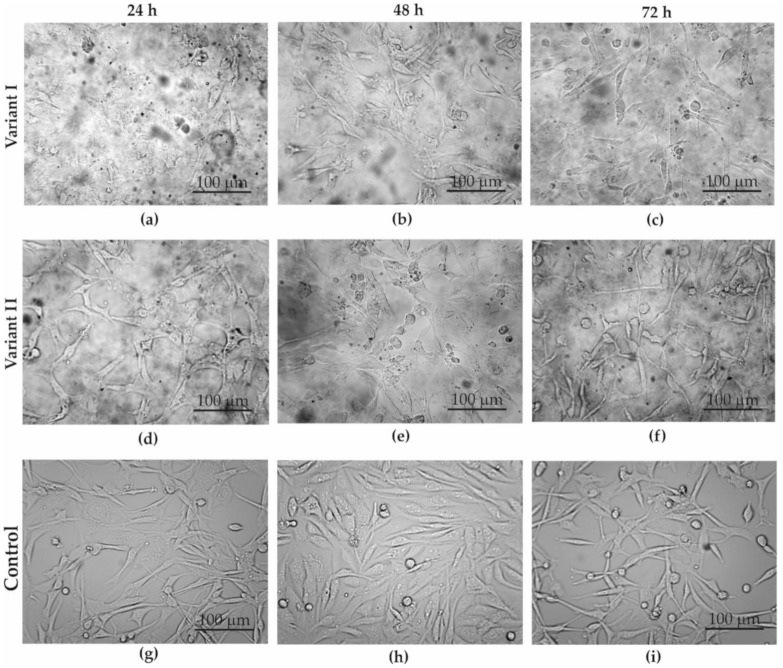
Optical microscope images of MDA-MB-231 cell culture in: (**a**–**c**) variant I; (**d**–**f**) variant II; (**g**–**i**) control; after: 24 (**a**,**d**,**g**), 48 (**b**,**e**,**h**), and 72 h (**c**,**f**,**i**).

**Figure 6:**
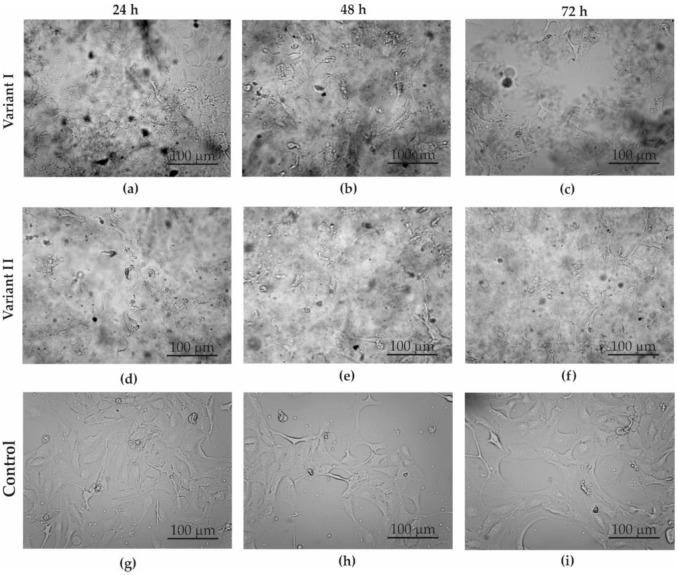
Optical microscope images of SW-954 cell culture in: (**a**–**c**) variant I; (**d**–**f**) variant II; (**g**–**i**) control; after: 24 (**a**,**d**,**g**), 48 (**b**,**e**,**h**), and 72 h (**c**,**f**,**i**).

The SW-954 cells were smaller and more compact than the MDA-MB-231 cells. In both cases (variant I—Figure 5a–c and Figure 6a–c; variant II—Figure 5d–f and Figure 6d–f), it was noticeable that there was a clear difference between the GO-treated cells and the control cells (control—Figure 5g–i, Figure 6g–i). In the case of variant I for the MDA-MB-231 cell line, we did not observe significant inhibition of cell count along with the increasing incubation time with GO as compared to the control. While a microscopic analysis of the SW-954 cell line treated with GO in variant I reveals a decreasing number of cells with increasing incubation time, this may indicate the effect of GO on the count of cancer cell lines. On the other hand, microscopic observation of the results from variant II indicated that GO slightly inhibits cells number and changes the morphology of SW-954 cells after 24, 48, and 72 h incubation, respectively, while no significant effect of GO on the cell count and morphology of MDA-MB-231 cells was noticed. Analysis of the cancer cell structure demonstrated a tendency towards a cytotoxic influence of graphene flakes on the cancer cell lines.

**Figure 7:**
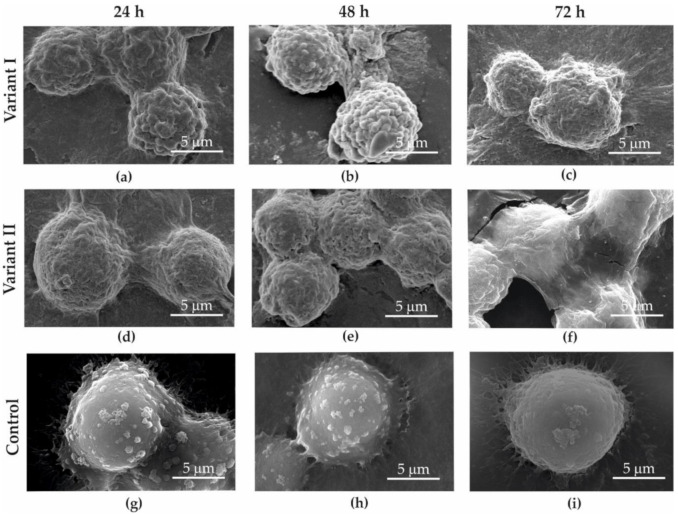
SEM image of MDA-MB-231 cancer cells obtained for variant I (**a**–**c**); variant II (**d**–**f**); control (**g**–**i**); after 24 (**a**,**d**,**g**), 48 (**b**,**e**,**h**), and 72 h (**c**,**f**,**i**).

**Figure 8:**
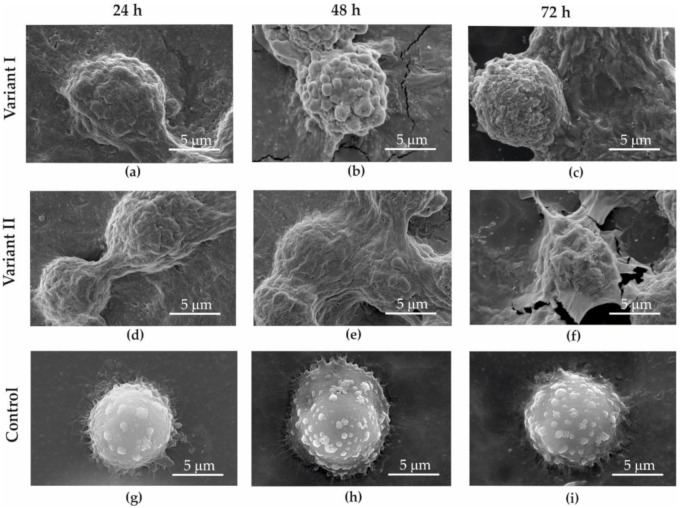
SEM image of SW-954 cancer cells obtained for variant I (**a**–**c**); variant II (**d**–**f**); and control (**g**–**i**); after 24 (**a**,**d**,**g**), 48 (**b**,**e**,**h**), and 72 h (**c**,**f**,**i**).

With a SEM analysis, it was observed that all cells treated with the GO aerosol (variant I—Figure 7a–c and Figure 8a–c; variant II—Figure 7d–f and Figure 8d–f) showed surficial deformation of the cell membrane.

While analyzing variant II, when the cancer cells were covered with GO, it was clearly observed that the graphene flakes precipitated on the surface of the cell membrane (Figure 8a). It was observed that right after deposition between the cell membrane and graphene (similar to the case of dead cancer cells), strong adhesive bonds cannot be observed. However, after 24–72 h of interaction with GO, cell morphology was characterized by constricted cell membranes (Figure 8b,c). GO adheres to the cells and covers them hermetically. After 72 h, the GO layer started to break due to strong adhesive forces between the cells and GO. Shrinking cells increased surface tension and caused GO cracks around cells (Figure 7f, Figure 8f).

Deformation of the cell membrane was not observed in control samples (control Figure 7g–i and Figure 8g–i), where GO did not interact with the examined cell lines.

Analysis of the SEM images showed that in variant II, a decrease in cell diameter and shrinking of the cell membrane was observed as a result of the GO interaction with cells. GO on the cell surface probably creates a barrier layer which cuts the cells off (partially or totally) from nutrients. It was also observed that after 72 h of incubation in variant II, the surface of GO began to crack and became loose. 

When coming into contact with cells, GO twines them and causes surface deformation. The degree of deformation might depend on the time of the interaction, cell age, and the number of graphene flakes influencing the cell membrane.

**Figure 9:**
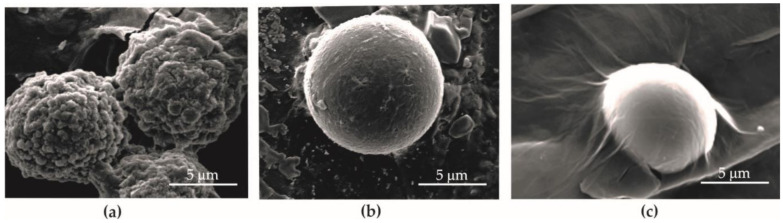
SEM images in variant I of MDA-MB-231 cancer cells with GO deposited on: (**a**) living cells and (**b**,**c**) dead.

It is very interesting that the changes in surface morphology of the cancer cells concern only the living cells. In the case where cells were already dead before GO deposition, no changes in cell morphology were observed. This was clearly visible with MDA-MB-231 cells (Figure 9).

SEM image analysis in variant I showed that the morphology of the cell membrane of the living cell with GO deposited on its surface (Figure 9a) differs in surface development as compared to the surface of cells that were dead before deposition or that died right after deposition (Figure 9b,c). Those cells showed a smooth surface under the GO layer. 

### 3.3. Cells Viability

**Figure 10:**
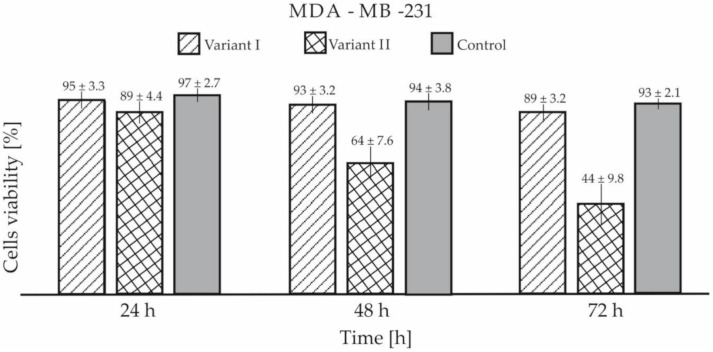
Cell viability of MDA-MB-231 in both tested variants.

**Figure 11:**
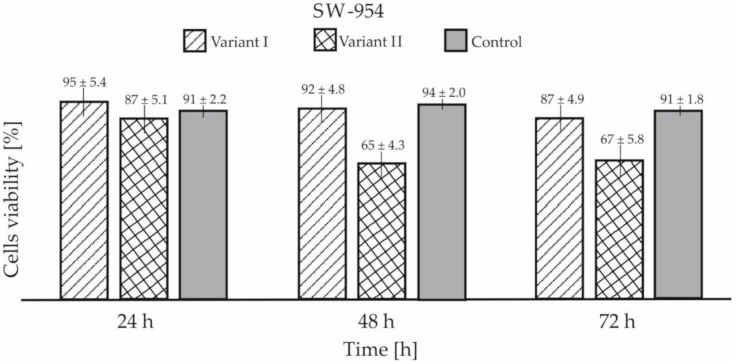
Cell viability of SW-954 in both tested variants.

A biological viability test with the use of trypan blue showed that cancers cells coated with GO were characterized by reduced viability compared to the control sample and cells cultured on a Petri dish coated with GO. The test showed reduced viability after 72 h in variant I for around 4.3% for both cell lines as compared to the control samples (Figure 10). For variant II, the differences in viability were significantly higher and were 52.7% for MDA-MB-231 and 26.4% for SW-954, respectively (Figure 11). The results presented in Figure 10 and Figure 11 are shown with standard deviation calculated from nine measurements.

## 4. Conclusions

In this paper presented, the influence of GO on the biological viability of triple-negative breast cancer and vulva cancer cells was examined. The manner of GO deposition during cell culturing was the factor that drew the authors’ attention. Two variants were examined: (I) cells were grown on a GO layer and (II) cells were covered with a GO layer. The results presented were compared to the control samples. The experiments were conducted in the same conditions (temperature, time of culturing, and culturing environments) but the location of the GO was the factor that had a crucial effect on cells. The viability of cells covered with GO (variant II) was lower for 52.7% for MDA-MB-231 and 26.4% for SW-954 lines as compared with the control samples. Cells cultured on a GO layer (variant I) showed a drop in viability of 4.3%, which suggests a scant effect of GO on the cells. This feature was similar for both tested cell lines. It is also possible that the GO layer acts as a barrier for trypan blue and does not allow the dye to react with cells. We cannot rule out the possibility that the viability results are lower than in reality and the cell count would be in fact higher. In addition, morphological changes among these cells were similar. Visible deformations of the shape of the cells were observed in samples with GO. At this point, it is difficult to assess if GO penetrates inside the cells or interacts with the cell membrane, thus impacting cell shape and viability. It is also possible that the drop in viability in variant II was an effect of GO covering the cells. GO, by adhering to the cell membrane, limited the passage of nutrients which caused a decrease in their viability.

It was observed that right after deposition (similar to the case of dead cancer cells) between the cell membrane and graphene, strong adhesive bonds cannot be observed. However, after 24–72 h of interaction with GO, cell morphology is characterized by constricted cell membranes.

GO deposition in the form of aerosol has never been performed before. Some of the issues that arose during the experiments presented require further investigation and will be tackled in the future. These will include the interaction of normal cells with GO aerosol. A comparison of the results of such experiments will allow it to be established whether a GO aerosol possesses anticancer properties. 

## Figures and Tables

**Table 1:** Petri dish surface roughness changes (mean out of 10 measurements) with standard deviation.

Surface Type	Mean Value Ra ^1^ [µm]	Mean Value Rz ^2^ [µm]
Petri dish surface	0.590 ± 0.04	2.88 ± 0.65
Petri dish surface after plasma cleansing	0.796 ± 0.14	3.25 ± 0.76
Petri dish surface after plasma cleansing and UV sterilization	0.690 ± 0.18	2.74 ± 0.22
Petri dish surface after plasma cleansing and GO deposition	0.848 ± 0.12	2.89 ± 0.28
Petri dish surface after plasma cleansing, GO deposition and vacuum drying	0.984 ± 0.32	4.14 ± 0.36
Petri dish surface after plasma cleansing, vacuum drying and UV sterilization	0.896 ± 0.29	3.85 ± 0.34

^1^ Ra—arithmetic average.^2^ Rz—maximum peak to valley height of the profile.
